# Supplementary material for: Cautionary lessons from the COVID-19 pandemic: Healthcare systems grappled with the dual responsibility of delivering COVID-19 and non-COVID-19 care
Source: PLOS Glob Public Health. 2024 Nov 4;4(11):e0002035. doi: 10.1371/journal.pgph.0002035 (PMC11534245; doi:10.1371/journal.pgph.0002035)
Supplement: Attachment — Submitted filename: Comments adressed_PLOS Global.docx [file pgph.0002035.s002.docx]

General comments

1. There are abbreviations used in the manuscript text that were not firstly defined before being used. Authors should address this observation.

- Modified.

2. Authors need to be consistent with the use of COVID-19, and not COVID

- Changed in full paper

Title

1. Need for the full title to be revised based on the number of words

- Revised

2. COVID in the title should be COVID-19

- Changed

Abstract

Findings to be changed to ‘’Results’’

- Changed

Introduction

1. Need for the introduction section to be reframed. The format used by the authors does not follow the normal flow of background information, magnitude of the problem, rationale or justification for the survey, and survey objectives.

- Modified to the suitability

Materials and Methods

1. This section needs to be well structured, with the sequence of study area, study design and duration, study population and eligibility criteria, sample size determination, sampling technique, data collection method(s), data analysis

- Modified to the suitability

2. There was no proper description of the survey area in terms of states and health facilities highlighted under the results section

- Description inserted.

461 hospitals from 18 states and 2 UTs of India responded to the survey.

3. How were the health facilities selected? Authors should state criteria used for selection of Public and Private Health facilities

- Stated the criteria used. Voluntary response sampling was adopted and non-responders were excluded. No specific allocation and selection of public and private hospitals was done

4. Authors to clearly state who the study population were, eligibility criteria used for selection?

- Study population - 20,347 hospitals registered on the National Health Authority (NHA) portal.

5. The survey sample size and how it was calculated? Was proportional allocation done for selection of respondents from public and private health facilities?

- Responded. No specific allocation and selection of public and private hospitals was done

6. Authors should highlight the sampling technique used for the survey.

- Mentioned: Voluntary Response Sampling

7. There was no information on public & private health facilities under the National Health Insurance Scheme in India

- Details of participated facilities is mentioned in results section.

8. There was no description of health care services provided under the National Health Insurance Scheme in India

- Current paper disseminates findings on the survey conducted and does not focus on the services provided under AB-PMJAY.

9. Information regarding urban hospitals to be completed before that of rural hospitals having poor internet

- Information regarding urban and rural hospitals is given as per their participation

10. ‘’In case of rural areas where the hospitals often experience poor internet connectivity, the research team helped them in filling up the form through telephone interviews’’. How did the researchers address response bias? For responses that did not meet the interviewers’ expectations, how was this handled? Were there some probing questions used during the interview?

- As the survey constituted closed ended, multiple option questions there was no scope of response bias or need for probing questions.

11. The type of data analysis done by authors not clearly illustrated. Level of statistical significance not documented.

- Mentioned with their significance in methodology and results.

12. ‘’We used logistic regression is used to understand the effect of hospital beds……’’. Delete ‘’is used’’

- Modified

13. Operational definitions should be included by authors

- Variables are defined.

14. Authors did not define what dependent (outcome) variables used, same for the independent (explanatory) variables

- All variables are defined.

15. ‘’In this dataset, we considered 18?? variables’’. Authors are to clarify this.

- Revised and clarified.

Results
1. It will be good that authors should include absolute numbers alongside proportions being reported under the results section

- Included.

2. Authors need to include the response rate as part of the first sentence in the first paragraph. (Section 3.1: State-wise distribution of the responses) Authors are to provide information on the sample size under the methods from which they will be able to provide information on the response rate

- Included.

- Considering the constraints and challenges of dealing with the start of the pandemic and to have representation from all states we fixed the ‘target sample size’ for responses as 5% of all hospitals from each state.

3. ‘’At least 5% of all empaneled hospitals under Ayushman Bharat Yojana were expected to respond from each state’’. This sentence under Lines 2-3, Section 3.1 supposed to be under Methods section.

- Moved to methods section

4. Authors should provide information on what constitute ‘’others (8.9%)’’ line 3 under the Section 3.2 Demographic characteristics of responding hospitals
- Others (8.9%) are minor teaching hospitals

5. Line 5 under Section 3.3 Changes in hospital services and availability of healthcare workers (‘’55.1% hospitals reported a decrease in number of OPD patients visiting…….). Authors used numbers to open a sentence, this should be corrected. Same for line 8 (‘’10.5% hospitals responded to the scarcity of nurses…) and line 13 (‘’49.5% were experiencing scarcity of staff in critical non-COVID areas such as intensive care units and dialysis units)

- Corrected

6. Section 3.3 Changes in hospital services and availability of healthcare workers Lines 11-13 (‘’It shows the extent of the scarcity of HCW in both private and public hospitals across all covered states’’) should be moved under the Discussion section.
- Moved

7. Authors need to provide information on COVID and non-COVID areas under the methods section

- Not a mandate of the study, hence not included.

8. Section 3.4 Determinants of hospital services. Authors provided information on bivariate analysis findings; however, this was not captured under the methods section.

- Included in methods.


Discussion
The authors at the opening of the discussion section should first provide overall summary findings of the survey by providing information that broadly answers the survey objectives.

- Summary given.
