# Supplementary material for: Cautionary lessons from the COVID-19 pandemic: Healthcare systems grappled with the dual responsibility of delivering COVID-19 and non-COVID-19 care
Source: PLOS Glob Public Health. 2024 Nov 4;4(11):e0002035. doi: 10.1371/journal.pgph.0002035 (PMC11534245; doi:10.1371/journal.pgph.0002035)
Supplement: Attachment — Submitted filename: Response to Reviewers_23082024.docx [file pgph.0002035.s007.docx]

To,

The Editors,

PLOS Global

Subject: Submission of Corrections in Article.

Dear Sir/Madam,

We have submitted our article for review and received feedback from the reviewers. We have addressed their suggested revisions and made the necessary corrections.

Thank you for your attention to this matter.

**Sub:** Explanation for queries raised on the article titled “Cautionary lessons from the COVID-19 Pandemic: Healthcare Systems Grappled with the dual responsibility of delivering COVID-19 and non-COVID-19 care. ”

**Reviewer #5:**

**Comment**: Consistency: the % (percentage) could be placed on the topmost row of the respective column of each table and not across each result on the table.

**Response**: Thank you for suggesting the relocation of the percentage values to the uppermost row of each corresponding column. This adjustment has been implemented on Page 12, Table 2, which focuses on the alterations in hospital services and the accessibility of manpower.

**Comment**: Figure 4 is not very clear i.e., what do the horizontal bar labels represent? For instance for the graph focused on beds > 250 does one represent those less than 250 and 1 those above it? Also, for private not-for-profits, what do 1 and 0 represent? All graphs miss labels on the vertical axis, please include them.

**Response**: All the bar plots were generated to show the number of hospitals in each category (“Yes” or “No”) for the significant categorical variables.

In the grouped bar plots, 0 denotes “No” and 1 denotes “Yes”. Labels have been changed on the vertical axis.

**Results:**

**Comment:** The statements, one: Large private for-profit hospitals were less likely to face staff shortages and second; private for-profit hospitals had significant staff reduction, contradict each other.

**Response:** Typo error (it should be large private not-for-profit hospitals)

**Comment:** It is also important for authors to provide, in the methods section, the hypothesis behind the choices of variables or associations. For example, what does high/low bed capacity represent or is it used as a proxy for hospital financial capital? Otherwise, how do we provide a linkage between bed capacity and the presence of separate COVID facility or increase and decrease in non-covid hospitalizations?

**Response:** The study by Gupta et al [1] found that patients who were admitted to hospitals with fewer than 50 ICU beds had a more than threefold higher risk of death than patients admitted to larger hospitals. [1] Gupta S, Hayek SS, Wang W, et al. Factors Associated With Death in Critically Ill Patients With Coronavirus Disease 2019 in the US. *JAMA Intern Med.* 2020;180(11):1436–1447. doi:10.1001/jamainternmed.2020.3596

**Comments on Discussion:**

**1. Reduce/avoid repeating the results. Instead, authors should focus on interpreting/showing implications and discussing the results.**

Response 1: Thank you for your perceptive remarks.

We appreciate your feedback on reducing the repetition of results and focusing more on the interpretation and implications in the research manuscript's Discussion section. Some of the changes are as follows:

In our nationwide survey, we observed a substantial decline in crucial hospital services. Notably, there was a 74.8% reduction in these services, with outpatient services experiencing a decline of 55.1%. Additionally, our findings revealed that only 66.4% of hospitals had accessible emergency services, while 56.2% of hospitals did not offer obstetric services.

Moreover, our investigation highlighted significant gaps in healthcare facilities, where 63.3% did not provide obstetric surgery services, and 44.5% could not perform emergency surgeries. We also noted a 22.6% reduction in elective surgeries.

Furthermore, our survey identified a critical staff shortage, with a 50.5% decrease in personnel across various roles, including administrative staff, nurses, and doctors. Intensive Care Unit (ICU) personnel saw a substantial reduction of 49.5%.

Lastly, we found an intriguing correlation in our study - hospitals performing emergency surgeries appeared to have a positive association with being covered under the PMJAY scheme. These findings raise important questions about the impact of healthcare policies and access to critical services during challenging times.

By emphasising these implications and interpretations, we aim to provide a deeper understanding of the significance of our results in the context of healthcare delivery and policy implications. Thank you once again for guiding us in improving the discussion section of our manuscript.

**2. Discussion partly entails showing and interpreting similarities and differences between the current study and others which were done elsewhere. This was hugely missing. Very few comparisons were made.**

Response 2: Thank you for your valuable feedback regarding the discussion section. Based on your recommendation, we have revised this section to include more detailed comparisons with previous studies. Some of the changes are as follows:

**Result**: In light of the insightful advice you provided, we have incorporated a comprehensive comparison of our findings with those of previous studies. Our nationwide survey reveals a substantial reduction in crucial hospital services, with a noteworthy 74.8% decrease in overall services, specifically a 55.1% decline in outpatient services. Moreover, access to emergency services was limited to only 66.4% of hospitals, and obstetric services were unavailable in 56.2% of healthcare facilities.

**Comparison**: We have identified relevant studies for comparison. For instance, S. Yamaguchi et al. demonstrated a significant decrease in hospitalizations and outpatient visits by 27% and 22%, respectively, during the middle of 2020 in Japan [7]. Farooq et al.'s study indicated a 59% reduction in hospital admissions and a 72% reduction in outpatient department (OPD) visits compared to the previous year's data [8]. Additionally, Yoon S et al. reported that only 55.5% of hospitals performed emergency procedures, and merely 22.6% of hospitals conducted elective surgeries [9].

**Result**: Our nationwide investigation also highlighted that 63.3% of healthcare facilities did not offer obstetric surgery services, and 44.5% did not provide emergency surgeries.

**Comparison**: To emphasize these findings, we have included comparisons with other studies. Elective surgeries witnessed a reduction of 22.6%. Helmy Abdelmalek et al.'s research pointed out a significant 55% decrease in the number of major surgeries performed within surveyed medical centres following the pandemic's declaration [10]. Furthermore, a study involving e-health records in the United Kingdom disclosed a 25.4% decrease in the count of emergency surgeries over two months [11]. The World Health Organization (WHO) reported that

44.5% of countries chose not to offer emergency surgeries, and 63.3% of countries lacked emergency obstetric and gynaecological surgical facilities, echoing our findings [12-13]. Additionally, a study by Cano-Valderrama et al. demonstrated a remarkable 65.4% decrease in emergency surgery activity in Spain [14].

**Result**: Our nationwide survey reveals a significant staff shortage of 50.5% across multiple roles, including administrative staff, nurses, and doctors, alongside a notable 49.5% reduction in Intensive Care Unit personnel.

**Comparison**: To provide a broader context, we now compare our findings to the study by Limb et al., which showed a decline in healthcare workers (HCWs) in physician and nursing care personnel categories [15]. They reported an overall decrease of 15.9%, 18.4%, 2.5%, and 9.8% among nursing staff, nursing aides, doctors, and other administrative staff, respectively. We also highlight the distinction that private for-profit hospitals experienced more significant staff attrition due to their smaller size and specialization [16]. Conversely, public hospitals and large private not-for-profit hospitals did not undergo substantial staff layoffs [17]. Smaller private hospitals with fewer than 50 beds were particularly affected, as they suffered losses due to suspended elective and emergency surgeries and difficulties in diagnosing and isolating COVID-19 patients [18-20].

**Result**: A significant majority of 61.2% of healthcare providers experienced heightened stress, anxiety, or fear while caring for patients during the COVID-19 pandemic, with 38.3% of HCWs stressed about viral exposure due to limited supplies of PPE and N95 masks.

**Comparison**: In response to your feedback, we have added more comparisons to support our findings. For instance, low-income patients with government (Medicaid) insurance had better access to healthcare, as revealed by Figueroa et al.'s study [38].

**Furthermore**, we acknowledge the significance of your input regarding the need for proper education and training of HCWs in pandemic preparedness. We have included references to studies that highlight the importance of such training to enhance expertise, skills, and mental well-being [25-26]. Notably, during the previous SARS outbreak in 2003, well-trained HCWs in infection control experienced the lowest stress levels [27]. This emphasizes the importance of preparedness in managing similar crises effectively.

We hope these revisions address your concerns and provide a more comprehensive discussion section that highlights the similarities and differences between our study and previous research. Your feedback has been invaluable in improving the quality of our manuscript.

3. **Some in-text citations needed to be included and should be included. At some points, citations were put at the end of sections which wholly contained the results of the current study. This is not proper and is unacceptable.**

Response 3: Thank you for your insightful advice and suggestions. Citations on the introduction section and discussion section were identified and modified.

4. **Ethical approval and IRB statements are the same and one should be dropped.** Response 4: Done

**Response to Reviewers**

General comments

1. There are abbreviations used in the manuscript text that were not first defined before being used. The authors should address this observation.

**-	Modified.**

2. Authors need to be consistent with the use of COVID-19, and not COVID

**-	Changed in full paper**

Title

1. Need for the full title to be revised based on the number of words
- **Revised**
  2. COVID in the title should be COVID-19
- **Changed**

Abstract

Findings to be changed to ‘’Results’’

- **Changed**

Introduction

1. Need for the introduction section to be reframed. The format used by the authors does not follow the normal flow of background information, the magnitude of the problem, the rationale or justification for the survey, and the survey objectives.

**-	Modified to the suitability**

Materials and Methods

1. This section needs to be well structured, with the sequence of study area, study design and duration, study population and eligibility criteria, sample size determination, sampling technique, data collection method(s), data analysis

**-	Modified to the suitability**

2. There was no proper description of the survey area in terms of states and health facilities highlighted under the results section **Description inserted.**

461 hospitals from 18 states and 2 UTs of India responded to the survey.

3. How were the health facilities selected? Authors should state the criteria used for the selection of Public and Private Health facilities

**- Stated the criteria used. Voluntary response sampling was adopted and non-responders were excluded. No specific allocation and selection of public and private hospitals was done**

4. Authors to clearly state who the study population were, and the eligibility criteria used for selection.

**- Study population - 20,347 hospitals registered on the National Health Authority (NHA) portal.**

5. The survey sample size and how it was calculated. Was proportional allocation done for the selection of respondents from public and private health facilities?

**- Responded. No specific allocation and selection of public and private hospitals was done**

6. Authors should highlight the sampling technique used for the survey.

**- Mentioned: Voluntary Response Sampling**

7. There was no information on public & private health facilities under the National Health Insurance Scheme in India

**- Details of participating facilities are mentioned in the results section.**

8. There was no description of health care services provided under the National Health Insurance Scheme in India

**The current paper disseminates findings on the survey conducted and does not focus on the services provided under AB-PMJAY.**

9. Information regarding urban hospitals to be completed before that of rural hospitals having poor internet

**- Information regarding urban and rural hospitals is given as per their participation**

10. ‘’In the rural areas where the hospitals often experience poor internet connectivity, the research team helped them fill out the form through telephone interviews’’. How did the researchers address response bias? For responses that did not meet the interviewers’ expectations, how was this handled? Were there some probing questions used during the interview?

**- As the survey constituted closed-ended, multiple-option questions there was no scope for response bias or need for probing questions.**

11. The type of data analysis done by the authors is not clearly illustrated. Level of statistical significance not documented.

**- Mentioned their significance in methodology and results.**

12. ‘’We used logistic regression to understand the effect of hospital beds……’’. Delete ‘’is used’’

**-	Modified**

13. Operational definitions should be included by the authors

**-	Variables are defined.**

14. The authors did not define what dependent (outcome) variables were used, same for the independent (explanatory) variables

**-	All variables are defined.**

15. ‘’In this dataset, we considered 18?? variables’’. The authors are to clarify this.

- **Revised and clarified.**

**Results**

1. It would be good that authors should include absolute numbers alongside proportions being reported under the results section

- **Included.**

2. Authors need to include the response rate as part of the first sentence in the first paragraph. (Section 3.1: State-wise distribution of the responses) Authors are to provide information on the sample size under the methods from which they will be able to provide information on the response rate

**-	Included.**

**- Considering the constraints and challenges of dealing with the start of the pandemic and to have representation from all states we fixed the ‘target sample size’ for responses as 5% of all hospitals from each state.**

3. ‘’At least 5% of all empanelled hospitals under Ayushman Bharat Yojana were expected to respond from each state’’. This sentence under Lines 2-3, Section 3.1 is supposed to be under the Methods section.

**-	Moved to the methods section**

4. Authors should provide information on what constitutes ‘’others (8.9%)’’ line 3 under the Section 3.2 Demographic characteristics of responding hospitals

**-	Others (8.9%) are minor teaching hospitals**

5. Line 5 under Section 3.3 Changes in hospital services and availability of healthcare workers (‘’55.1% hospitals reported a decrease in number of OPD patients visiting…….). The authors used numbers to open a sentence, this should be corrected. Same for line 8 (‘’10.5% hospitals responded to the scarcity of nurses…) and line 13 (‘’49.5% were experiencing a scarcity of staff in critical non-COVID areas such as intensive care units and dialysis units)

**-	Corrected**

6. Section 3.3 Changes in hospital services and availability of healthcare workers Lines 11-13

(‘’It shows the extent of the scarcity of HCW in both private and public hospitals across all covered states’’) should be moved under the Discussion section.

- **Moved**

7. Authors need to provide information on COVID and non-COVID areas under the methods section

- **Not a mandate of the study, hence not included.**

8. Section 3.4 Determinants of hospital services. The authors provided information on bivariate analysis findings; however, this was not captured under the methods section.

**-	Included in methods.**

**Discussion**

The authors at the opening of the discussion section should first provide an overall summary findings of the survey by providing information that broadly answers the survey objectives.

- **Summary given.**

**Review comments to Authors**

There is still one area that must be looked at thoroughly, the discussion. Authors have, in most of this section, repeated the results. Instead, they are required to provide an interpretation and implication of their findings. **Response: Necessary changes are incorporated as suggested by the reviewer.**

**Very minor issues to address:**

**Comment :** To be consistent on the use of the words COVID-19; there are some paragraphs which still contain the wrong word COVID without the suffix -19

**Response**: Necessary changes have been incorporated as suggested by the reviewer, as reflected in the previously uploaded manuscript. We do not understand the need for any further updates at this time and would appreciate guidance on any additional modifications required.

**Comment :** While it is commendable to include as many comparative studies as possible in the discussion, it get a little bit annoying to add tables showing methods and results of these studies. I would suggest to retain texts and add information pertaining to the implications of their findings or discrepancies between the studies.

**Response**: The tables provided exclusively contain data from our study. No tables incorporating data from external studies have been included. Nevertheless, we would be grateful for any identification of discrepancies on our part, so that we may make the necessary modifications**.**

**Comment** : The following sentence from a paragraph in the methods "hospitals during May – July 202" has a wrong year. Please correct that.

**Response:** This change has been implemented previously. We believe that this error is not present in our updated manuscript; however, we would be grateful if it could be addressed should it be identified by the reviewer.

**Comment** : Changes to reference list-

**Response** : No alterations are to be made to the reference list at this time. However, if there are any specific references that you find to be insufficient or inadequate, please provide us with guidance on these matters, as we are unable to address this on our own.

Regards,

On behalf of all authors
