# Supplementary material for: Cautionary lessons from the COVID-19 pandemic: Healthcare systems grappled with the dual responsibility of delivering COVID-19 and non-COVID-19 care
Source: PLOS Glob Public Health. 2024 Nov 4;4(11):e0002035. doi: 10.1371/journal.pgph.0002035 (PMC11534245; doi:10.1371/journal.pgph.0002035)
Supplement: Attachment — Submitted filename: Reviewer comments 10 Jan.docx [file pgph.0002035.s004.docx]

Reviewer #5:

**Comment**: Consistency: the % (percentage) could be placed on the topmost row of respective column of each table and not across each result on the table.

**Response**: Thank you for pointing out the idea of relocating the percentage values to the uppermost row of each corresponding column. This adjustment has been implemented in Page 12, Table 2, which focuses on the alterations in hospital services and the accessibility of manpower.

**Comment**: Figure 4 is not very clear i.e., what do the horizontal bar labels represent? For instance for the graph focused on beds > 250 does one represent those less than 250 and 1 those above it? Also, for private not for profit, what does 1 and 0 represent. All graphs miss labels on the vertical axis, please include them.

**Response**: All the bar plots were generated to show the number of hospitals in each category (“Yes” or “No”) for the significant categorical variables.

In the grouped bar plots 0 denotes “No” and 1 denotes “Yes”. Labels has been changed on the vertical axis.

**Results:**

**Comment:** The statements, one: Large private for profit hospitals were less likely to face staff shortages and second; private for profit hospitals had significant staff reduction, are contradicting each other.

**Response:** Public hospitals and large hospitals (Public and private) were less likely having staff shortage (Fig 5a) but less than 250 bedded smaller hospital (Public or private) were more likely to experienced shortage in staff because of significant reduction of revenue (Fig 5b).

**Comment:** It is also important for authors to provide, in the methods section, the hypothesis behind the the choices of variables or associations For example, does high/low bed capacity represent or is it used as a proxy for hospital financial capital? Otherwise, how do we provide a linkage between bed capacity and presence of separate Covid facility or increase and decrease in non-covid hospitalizations?

**Response:** The study by Goldberg [1] found that patients who were admitted to hospitals with fewer than 50 ICU beds had a more than threefold higher risk of death than patients admitted to larger hospitals.

[1] Goldberg, C. (2020, July 15). In Smaller Hospitals, COVID-19 Patients At Triple The Risk Of Dying, National Study Finds | WBUR News. WBUR.org. https://www.wbur.org/news/2020/07/15/covid-hospital-mortality-death-bigger

**Comments on Discussion:**

**1. Reduce/avoid repeating the results. Instead authors should focus to interpret/show implication and discuss the results.**

Response 1: Thank you for your insightful advice and suggestions. We appreciate your feedback on reducing the repetition of results and focusing more on the interpretation and implications in the research manuscript's Discussion section. Some of the changes are as follows:

In our nationwide survey, we observed a substantial decline in crucial hospital services. Notably, there was a 74.8% reduction in these services, with outpatient services experiencing a decline of 55.1%. Additionally, our findings revealed that only 66.4% of hospitals had accessible emergency services, while 56.2% of hospitals did not offer obstetric services.

Moreover, our investigation highlighted significant gaps in healthcare facilities, where 63.3% did not provide obstetric surgery services, and 44.5% lacked the capacity to perform emergency surgeries. We also noted a 22.6% reduction in elective surgeries.

Furthermore, our survey identified a critical staff shortage, with a 50.5% decrease in personnel across various roles, including administrative staff, nurses, and doctors. Intensive Care Unit (ICU) personnel saw a substantial reduction of 49.5%.

Lastly, we found an intriguing correlation in our study - hospitals performing emergency surgeries appeared to have a positive association with being covered under the PMJAY scheme. These findings raise important questions about the impact of healthcare policies and access to critical services during challenging times.

By emphasizing these implications and interpretations, we aim to provide a deeper understanding of the significance of our results in the context of healthcare delivery and policy implications. Thank you again for guiding us in improving the discussion section of our manuscript.

**2. Discussion partly entails showing and interpreting similarities and differences of the current study and others which were done elsewhere. This was hugely missing. Very few comparisons were made.**

Response 2: Thank you for your valuable feedback regarding the discussion section. Based on your suggestion, we have revised this section to include more detailed comparisons with previous studies. Some of the changes are as follows:

Result: In light of the insightful advice you provided, we have incorporated a comprehensive comparison of our findings with those of previous studies. Our nationwide survey reveals a substantial reduction in crucial hospital services, with a noteworthy 74.8% decrease in overall services, specifically a 55.1% decline in outpatient services. Moreover, access to emergency services was limited to only 66.4% of hospitals, and obstetric services were unavailable in 56.2% of healthcare facilities.

Comparison: We have identified relevant studies for comparison. For instance, S. Yamaguchi et al. demonstrated a significant decrease in hospitalizations and outpatient visits by 27% and 22%, respectively, during the middle of 2020 in Japan [7]. Farooq et al.'s study indicated a 59% reduction in hospital admissions and a 72% reduction in outpatient department (OPD) visits compared to the previous year's data [8]. Additionally, Yoon S et al. reported that only 55.5% of hospitals performed emergency procedures, and merely 22.6% of hospitals conducted elective surgeries [9].

Result: Our nationwide investigation also highlighted that 63.3% of healthcare facilities did not offer obstetric surgery services, and 44.5% did not provide emergency surgeries.

Comparison: To emphasize these findings, we have included comparisons with other studies. Elective surgeries witnessed a reduction of 22.6%. Helmy Abdelmalek et al.'s research pointed out a significant 55% decrease in the number of major surgeries performed within surveyed medical centers following the pandemic's declaration [10]. Furthermore, a study involving e-health records in the United Kingdom disclosed a 25.4% decrease in the count of emergency surgeries over a two-month period [11]. The World Health Organization (WHO) reported that 44.5% of countries chose not to offer emergency surgeries, and 63.3% of countries lacked emergency obstetric and gynecological surgical facilities, echoing our findings [12-13]. Additionally, a study by Cano-Valderrama et al. demonstrated a remarkable 65.4% decrease in emergency surgery activity in Spain [14].

Result: Our nationwide survey reveals a significant staff shortage of 50.5% across multiple roles, including administrative staff, nurses, and doctors, alongside a notable 49.5% reduction in Intensive Care Unit personnel.

Comparison: To provide a broader context, we now compare our findings to the study by Limb et al., which showed a decline in healthcare workers (HCWs) in physician and nursing care personnel categories [15]. They reported an overall decrease of 15.9%, 18.4%, 2.5%, and 9.8% among nursing staff, nursing aides, doctors, and other administrative staff, respectively. We also highlight the distinction that private for-profit hospitals experienced more significant staff attrition due to their smaller size and specialization [16]. Conversely, public hospitals and large private not-for-profit hospitals did not undergo substantial staff layoffs [17]. Smaller private hospitals with fewer than 50 beds were particularly affected, as they suffered losses due to suspended elective and emergency surgeries and difficulties in diagnosing and isolating COVID-19 patients [18-20].

Result: A significant majority of 61.2% of healthcare providers experienced heightened stress, anxiety, or fear while caring for patients during the COVID-19 pandemic, with 38.3% of HCWs stressed about viral exposure due to limited supplies of PPE and N95 masks.

Comparison: In response to your feedback, we have added more comparisons to support our findings. For instance, low-income patients with government (Medicaid) insurance had better access to healthcare, as revealed by Figueroa et al.'s study [38].

Furthermore, we acknowledge the significance of your input regarding the need for proper education and training of HCWs in pandemic preparedness. We have included references to studies that highlight the importance of such training to enhance expertise, skills, and mental well-being [25-26]. Notably, during the previous SARS outbreak in 2003, well-trained HCWs in infection control experienced the lowest stress levels [27]. This emphasizes the importance of preparedness in managing similar crises effectively.

We hope these revisions address your concerns and provide a more comprehensive discussion section that highlights the similarities and differences between our study and previous research. Your feedback has been invaluable in improving the quality of our manuscript.

**3. Some intext citations were missing and should be included. At some points, citations were put at the end of sections which wholly contained the results of the current study. This is not proper and is unacceptable.**

Response 3: Thank you for your insightful advice and suggestions. Citations on the introduction section and discussion section were Identified and Modified.

**4. Ethical approval and IRB statements are the same and one should be dropped.**

Response 3: Done
